# California—Her Hospitals—Prevalent Diseases—Mortality, &c.

**Published:** 1852-07

**Authors:** 


### ECLECTIC DEPARTMENT,

### AND SPIRIT OF THE MEDICAL PERIODICAL PRESS.

### *California—Her Hospitals—Prevalent Diseases—Mortality, etc.*

Through the kindness of a friend, we have recently received the Report of the Trustees and Physicians of the *Sacramento State Hospital,* from which we glean the subjoined interesting facts and statistics:

The hospital was opened for the reception of patients on the 28th of May, 1851, and consequently had been in operation up to the 10th January, 1852, when this report was made out, seven months and thirteen days. During these seven months and thirteen days

| | | |
|---|---|---|
| The whole number admitted was | 592 | |
| " " discharged | 415 | |
| " " died | 72 | |
| Remaining in Hospital | | 104 |
| For the same time there were admitted in Insane Department | | 38 |
| Discharged cured | | 16 |
| Remaining in hospital | | 22 |
| Received from San Francisco | | 20 |

We append some of the principal diseases for which the 592 patients were admitted, in order to indicate the influence of climate upon emigrants:

Admitted of bilious remittent fever, 123; of rheumatism, 49; of intermittent fever, 46; of typhoid fever, 33; do. mental derangement, 38; do. diarrhœa, 38; do. wounds of various kinds, 30; do. Panama fever, 23; do. erysipelas, 11; and scorbutis, 11.

The foregoing seems to be the prevalent diseases for which patients were admitted into the Sacramento State Hospital. The other diseases which go to complete the list, are such as are met with in our hospitals in this portion of the United States, and therefore deserve no special notice.

Of the 592 admissions 72 died, and of the following diseases:

Dysentery, 8; abscess, 1; consumption, 5; diarrhœa, 14; bronchitis, 4; enteritis, 2; cerebritis, 4; scorbutis, 1; hemiphlegia, 1; erysipelas, 2; anasarca, 1; coxalgia, 2; fever congestive, 2; fever bilious remittent, 5; scarlatina, 1; bowels, ulceration of, 1; fever typhoid, 13; fever Panama, 4; delirium tremens, 2—making a total of 72 deaths out of 592 admissions.

Of the admissions, 342 were natives of the United States; and 250 adopted citizens, representing 20 different foreign countries.

The report concludes in the following words—as drawn up by Doctors Bryarly and Williams, the former the visiting, and the latter the resident physician, of the hospital:

"By referring to our report of the different diseases, it is easy to observe what may be considered as the prevailing diseases in this, the northern district of California.

"The largest number of any one class have been those of fever, particularly those of bilious remittent fever. This is not surprising, when it is considered that every feature of the country, and the general habits of the people are most conducive to this disease. During the dry season, the miners are compelled to resort to the rivers and water courses for work; here they are exposed to all the miasma originating from the decomposition of the vegetable matter from the overflown lands during the wet season. This produces intermittent, or common chills and fever.

"The general living of these people is decidedly bad; not only in reference to their food, but more particularly in reference to their sleeping apartments. They either sleep in the open air, exposed to the sudden changes peculiar to our climate, or they are huddled into tents and cabins, where they cannot but suffer from the effects of contaminated atmosphere. These things, connected with the fact that many of them work in the water six or eight hours each day, bring about such a state of the system, and such a habit of body, as renders it peculiarly susceptible to take on the most malignant forms of every disease with which they are attacked. It is thus that our worst fevers are produced, and these are the reasons of their frequency in our district.

"Although bilious remittent fever has far doubled the number of any other fever, it is to be observed that the mortality has been much less in comparison.

"From our mortality report, the greatest number of deaths have been from chronic diarrhœa. This is the most formidable disease in our whole country. We find it mostly attacking those recent in the country, and almost always following the extreme debility of the acclimating fever, Panama, typhus or ship fever, to which it seems to be the most regular sequel. The neglect of this in its acute stage, is followed by its passing into the chronic. The fact that there are so many existing causes, such as strong mental emotion, of a depressing or anxious kind, exposure to dampness and cold, indigestible food, intoxicating drinks, bad water, and general debility, that oftener the organic disease, before coming under the treatment of a physician, is so great, as to be out of the reach of human aid.

"In some of the northern portions of this district, during the past summer, the erysipelas has raged as an epidemic, with great mortality; and in some few places, even now, continues its ravages.

"The counties of Shasta, Nevada, and El Dorado, have been the worst sufferers. In many instances every inhabitant of small mining camps has been attacked, often assuming the most malignant form and proving fatal in a very few hours.

"The most apparent cause seems to be, the peculiar constitution of the atmosphere, exposure to all weathers, bad and unwholesome living, general tendency to scorbutis, which, combined with a natural predisposition, and the contaminated air of crowded and ill-ventilated apartments, are all calculated to render the subject peculiarly susceptible to erysipelatous inflammation.

"It will be seen, that although situated in the interior, we have not been exempt from the reception of 'Panama fever' in our wards.

"From the last steamer that arrived, (the Northerner) fourteen of her passengers have been admitted here.

"The appellation of 'Panama fever,' is very common at the present day; but the cases from the Northerner can be much more easily recognized under the head of 'ship fever.'"—*New Orleans Med. and Surg. Journal.*

